# Dosimetric effects of adaptive prostate cancer radiotherapy in an MR-linac workflow

**DOI:** 10.1186/s13014-020-01604-5

**Published:** 2020-07-10

**Authors:** Annika Mannerberg, Emilia Persson, Joakim Jonsson, Christian Jamtheim Gustafsson, Adalsteinn Gunnlaugsson, Lars E. Olsson, Sofie Ceberg

**Affiliations:** 1 Department of Medical Radiation Physics, Lund University, Lund, Sweden; 2 Department of Hematology, Oncology and Radiation Physics, Skåne University Hospital, Lund, Sweden; 3 Medical Radiation Physics, Department of Translational Medicine, Lund University, Malmö, Sweden; 4 Department of Radiation Sciences, Umeå University, Umeå, Sweden

**Keywords:** Motion induced dose effects, MR-linac, Prostate radiotherapy, Intrafractional motion

## Abstract

**Background:**

The purpose was to evaluate the dosimetric effects in prostate cancer treatment caused by anatomical changes occurring during the time frame of adaptive replanning in a magnetic resonance linear accelerator (MR-linac) workflow.

**Methods:**

Two MR images (MR1 and MR2) were acquired with 30 min apart for each of the 35 patients enrolled in this study. The clinical target volume (CTV) and organs at risk (OARs) were delineated based on MR1. Using a synthetic CT (sCT), ultra-hypofractionated VMAT treatment plans were created for MR1, with three different planning target volume (PTV) margins of 7 mm, 5 mm and 3 mm. The three treatment plans of MR1, were recalculated onto MR2 using its corresponding sCT. The dose distribution of MR2 represented delivered dose to the patient after 30 min of adaptive replanning, omitting motion correction before beam on. MR2 was registered to MR1, using deformable registration. Using the inverse deformation, the structures of MR1 was deformed to fit MR2 and anatomical changes were quantified. For dose distribution comparison the dose distribution of MR2 was warped to the geometry MR1.

**Results:**

The mean center of mass vector offset for the CTV was 1.92 mm [0.13 – 9.79 mm]. Bladder volume increase ranged from 12.4 to 133.0% and rectum volume difference varied between −10.9 and 38.8%. Using the conventional 7 mm planning target volume (PTV) margin the dose reduction to the CTV was 1.1%. Corresponding values for 5 mm and 3 mm PTV margin were 2.0% and 4.2% respectively. The dose to the PTV and OARs also decreased from D1 to D2, for all PTV margins evaluated. Statistically significant difference was found for CTV D_min_ between D1 and D2 for the 3 mm PTV margin (*p* < 0.01).

**Conclusions:**

A target underdosage caused by anatomical changes occurring during the reported time frame for adaptive replanning MR-linac workflows was found. Volume changes in both bladder and rectum caused large prostate displacements. This indicates the importance of thorough position verification before treatment delivery and that the workflow needs to speed up before introducing margin reduction.

## Background

Prostate cancer is the second most prevalent cancer diagnosis in men worldwide [1]. One common treatment approach is external beam radiotherapy (EBRT). This can be the sole treatment or could be combined with surgery or hormonal therapy [2]. The standard fractionation for EBRT of localized prostate cancer has long been 1.8 – 2.0 Gy per fraction to a total dose of 74 – 78 Gy [3]. However, studies show that hypo- or ultra-hypofractionated prostate radiotherapy achieves similar tumour control without increased late side effects [3–5]. One regime, which currently is used in our clinic, has decreased the number of fractions, from 39 to 7, using a fractionation dose of 6.1 Gy. A normal distribution will be obtained if the delivered dose distribution is averaged over a total of 39 fractions, despite motion displacements [6]. However, this is not necessarily the case when the dose is delivered using 7 fractions. For 7 fractions, the dosimetric effects of motion displacement of the tumour during one fraction can have a substantial impact, irradiating surrounding healthy tissue with a high dose and the remaining fractions may not be enough to compensate for the underdosage of the tumour.

To compensate for uncertainties a margin is added to the tumour. There are many recipes for margin estimation available. Using the most frequently applied method, originally introduced by van Herk et al. [6], a common used planning target volume (PTV) margin for conventional radiotherapy of localized prostate cancer is 7 mm [7]. For ultra-hypofractionation a PTV margin of 5 mm has also been used [8].

One option for decreasing the PTV margin is to eliminate the systematic deviation that arises during the co-registration of the computed tomography (CT) and magnetic resonance (MR) images [9, 10]. Nyholm et al. [10] stated that the systematic uncertainty of co-registration of the CT and MR is 2 – 3 mm. This uncertainty has resulted in the development of the MR imaging (MRI) only workflows. In MRI-only, all delineation and treatment planning are performed based on the MR images and a synthetic CT (sCT) is generated only for dose calculation purposes [9]. The clinical implementation of MRI-only has been studied in our clinic [11].

Intrafractional prostate motion is a well-known phenomenon [12–19] and can have dosimetric impact. Nejad-Davarani et al. [19] found a 20.2% decrease in the dose to 95% of the volume (D_95%_) in the PTV due to prostate displacement occurring during approximately 45 minutes (min). There are various reasons for prostate displacements, such as bladder filling, rectum activity and patient motion due to muscle tension or leg motion [12]. The prostate can be displaced by more than 10 mm during a conventional radiotherapy treatment session [14, 15, 17], and the nature of the motion is often a combination of large, sudden shifts and a slow drift [15]. It has also been shown that the probability of motion increases with time [13–19], mainly in the longitudinal and vertical direction [14–16, 18, 19] .

One relatively new method in radiotherapy for managing prostate motion is using online MR guidance. An MR linear accelerator (MR-linac) offer the possibility of daily adaptive replanning [20–23]. A 3 mm PTV margin for the prostate has been suggested and used in recent studies [23–25], as a result of the ability to adapt the dose distribution on a daily basis and perform imaging with high soft tissue contrast during beam delivery. However, the adaptive replanning process of the MR-linac can be considerably time-consuming compared to the duration of a fraction delivered with a conventional linac. Provided that no problems are encountered in the process, time spans between 20 and 40 min, from daily MR acquisition to beam on have been reported for MR-linac workflows [20–23]. This implies that the patient is treated with a plan adapted according to the anatomy valid up to 40 min earlier. Taking the increased probability of prostate displacement with respect to time into consideration, there might be a risk of adverse dosimetric effects treating prostate cancer patients using the MR-linac workflow. This risk is especially prominent for hypofractionation using small PTV margins.

The purpose of this study was to evaluate the dosimetric effects on a single fraction that arise from anatomical changes and prostate displacement occurring during the time frame corresponding to the daily adaptive replanning process for prostate cancer patients in the MR-linac workflow. Prostate shifts and anatomical changes, such as difference in volume, were quantified. This was achieved by acquiring MR images on a conventional MR scanner.

## Methods

In total, 35 prostate cancer patients were enrolled in the study. All patients had localized prostate cancer and were treated between March 2017 and July 2018, according to an MRI-only radiotherapy workflow. This workflow was part of a study performed at the radiotherapy department at Skåne University Hospital, Lund, approved by the ethical review board in Lund, Sweden (No. 2016/1033) [9]. All patients included in the present study had therefore undergone MR examination prior treatment planning. MR imaging was performed using a GE Discovery 3.0 T 750w (Software version DV25.1-R02–1649.a, GE Healthcare, Chicago, Illinois, USA) with the patients positioned on a flat tabletop with a 16 channel GE GEM Anterior Array coil placed on stiff coil bridges to avoid deforming the body contour of the patients. The knees and legs were fixated using a Combifix™ (Civco Radiotherapy), which was also used for fixation during treatment.

During MR imaging, two large field of view (FOV) T2-weighted MR images (MR1 and MR2) were acquired, separated by 30 min with the patient in treatment position during the complete imaging examination. This time is in accordance with the adaptive replanning time in an MR-linac system [20–23]. sCT images (MriPlanner™, Spectronic Medical, Helsingborg, Sweden) were generated for both MR images (sCT1 for MR1 and sCT2 for MR2). Delineation of the clinical target volume (CTV) and organs at risk (OARs), including rectum, bladder and genitals, was performed based on MR1 by the same physician for all patients. For each patient, three ultra-hypofractionated 6 MV flattening filter free (FFF) volumetric modulated arc therapy (VMAT) treatment plans with a total dose of 42.7 Gy, in 7 fractions, were created for sCT1. The first treatment plan was optimised using the clinically used PTV margin of 7 mm. In the second plan a 5 mm PTV margin was applied to investigate a margin frequently used for ultra-hypofractionation. For the third plan a PTV margin of 3 mm was used, consistent with the margins used for prostate cancer patients treated with MR-linac [23–25].

**Fig. 1:**
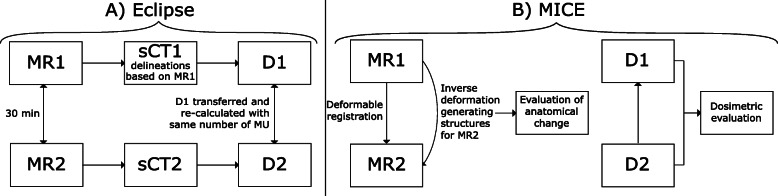
Schematic illustration of the workflow used for evaluating anatomical change and dosimetric effects between first (D1) and second (D2) dose distribution

The ultra-hypofractionated plans were created by the same medical physicist and optimised to meet the local clinical dose volume histogram (DVH) criteria (Table 1). The treatment planning system (TPS) used was Eclipse with the algorithm of Eclipse v.13.6.23 used for all optimisations. The plans consisted of two full arcs and the dose distribution was normalised so that 100% of the prescribed dose covered 50% of the PTV. The plan generating the first dose distribution, D1, was recalculated onto sCT2 with the same number of monitor units (MU) and field setup, creating a second dose distribution, D2 (Fig. 1). D2 corresponded to the dose that the patient would receive if 30 min were spent on daily adaptive replanning after initial imaging, omitting correction for motion occurring during this time before the radiation delivery. The dosimetric effect of patient motion was evaluated for a single fraction. The accumulated dose from all fractions is the most relevant, however in this study that would have meant assuming the same motion pattern for all 7 fractions, which was not considered reasonable, since the probability for the patient to move in the exact same pattern the subsequent fractions is low.

**Table 1:** Dose volume histogram criteria used during treatment planning

Volume	Criterion	Criterion, 1 fraction
CTV	D_min_ ≥ 41.5 Gy	D_min_ ≥ 5.93 Gy
PTV	D_95%_ ≥ 41.1 Gy	D_95%_ ≥ 5.87 Gy
Rectum	D_15%_ ≤ 38.0 Gy	D_15%_ ≤ 5.43 Gy
PTV	D_98%_ ≥ 40.6 Gy	D_98%_ ≥ 5.80 Gy
Rectum	D_10%_ ≤ 41.0 Gy	D_10%_ ≤ 5.86 Gy
Bladder	D_mean_ ≤ 34.0 Gy	D_mean_ ≤ 4.86 Gy

The DVH criteria to be achieved for a prostate cancer patient. The middle column shows the criteria used for optimisation and the right column shows the corresponding criteria for 1 fraction.

To quantify the anatomical changes and to compare D1 and D2, deformable registration and dose warping were carried out using MICE Toolkit v.1.0.9 (NONPI Medical, Umeå, Sweden). The MR images and corresponding dose distributions were exported from the TPS and imported into MICE Toolkit, where there is the option to use Elastix [26, 27] for image registration. MR2 was deformably registered to MR1 and the inverse deformation was applied to the clinical structures delineated on MR1, generating structures for MR2. The outer contour of the bladder was manually delineated in MR1 and MR2 and were used to guide the deformable image registration. Anatomical changes and motion that had occurred in the patient between the image acquisitions could thereby be quantified. The volume difference for rectum and bladder was assessed and the motion of the CTV was determined by extracting the center of mass (CoM) position of the CTV in both MR1 and MR2.

To enable DVH comparison between D1 and D2 using the structures created on MR1, the dose distributions had to be in the same geometry. D2, containing no structures, was warped to the geometry of D1, containing all structures. This was performed using the deformation field from the registration of MR2 to MR1. Before the dose warp D2 was essentially D1 calculated on another sCT. With dose warping, D2 corresponded to the absorbed dose delivered to the patient after 30 min, anatomical changes included. The deformed dose map D2 was compared to the original dose distribution D1, using the original structures (Fig. 1). The quality of deformable registration was controlled visually. To assure that no structure had folded on top of itself in the deformable registration the Jacobian was evaluated in MICE Toolkit with no negative values for the Jacobian obtained.

Wilcoxon signed-rank test, with a significance level of 0.05, was used to evaluate if there was a statistically significant difference between the dose to the CTV, PTV, rectum and bladder in D1 and D2. This was done for the dose distributions with PTV margin of 7 mm, 5 mm and 3 mm, respectively.

## Results

**Fig. 2:**
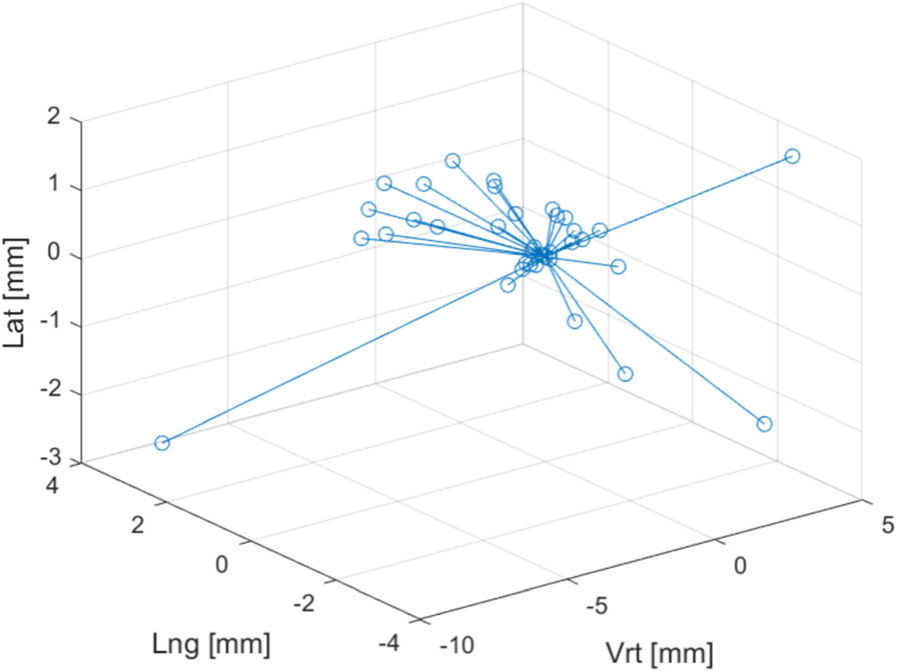
The CTV center of mass (CoM) offset from MR1 to MR2 for each patient. The circles indicate an individual CoM displacement and the lines all derive from the origin. Positive values correspond to a CTV offset in the anterior, cranial or right direction

**Fig. 3:**
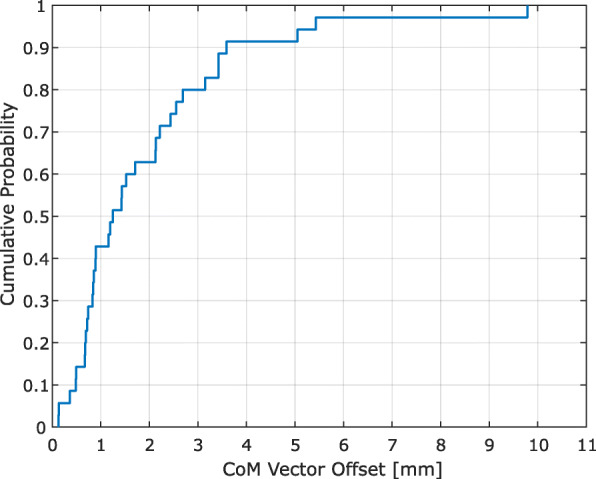
Cumulative probability histogram for the CTV center of mass (CoM) vector offset. A vector displacement of 3 mm or less was seen for 80% of the study population

Analysis of prostate motion occurring during 30 min between imaging showed that the mean CoM offset for the CTV was −0.49 mm [−9.08 – 4.23 mm], −0.32 mm [−2.89–2.75 mm] and 0.11 mm [−2.44 – 1.81 mm] in the anterior-posterior (AP), cranio-caudal (CC) and left-right (LR) direction respectively (Fig. 2). Positive values indicate a motion in the anterior, cranial or right direction. The mean CoM vector offset for the CTV was 1.92 mm with a range of 0.13 – 9.79 mm. Prostate motion was generally seen in the AP and CC directions, with largest motion in the AP direction. Anterior prostate displacement occurred mainly due to rectal gas appearing in MR2, forcing the prostate anteriorly. Prostate motion in the posterior direction was caused mainly by the patient relaxing from MR1 to MR2. For some patients, the bladder also seemed to shift the prostate posteriorly. Patient #27 had the largest prostate displacement. This motion was due to the patient clenching his gluteal muscles in MR1 and then relaxing in MR2, causing a large posterior shift of the prostate. The probability of a prostate displacement of 3 mm or more during 30 min was 20%. The probability of a ≥ 7 mm displacement was 2.9% (Fig. 3).

The bladder volume increased from MR1 to MR2 on average with 40.9% [12.4 – 133.0%]. The rectum volume difference varied between −10.9% and 38.8%. Negative differences of the rectum volume were mainly caused by rectum gas or filling present at MR1 acquisition, which passed the prostate area before MR2 was acquired.

**Fig. 4:**
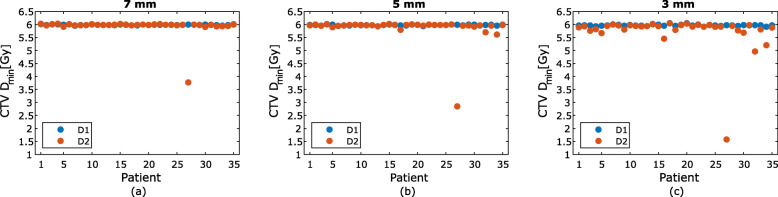
The CTV D_min_ dose for the dose distributions D1 (blue) and D2 (orange) for PTV margins 7 mm (**a**), 5 mm (**b**) and 3 mm (**c**). Patient #27 had the largest prostate displacement and had therefore also the largest reduction in CTV D_min_

**Fig. 5:**
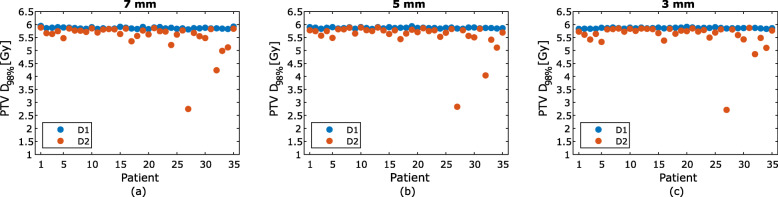
The PTV D_98%_ dose for the dose distributions D1 (blue) and D2 (orange) for PTV margins 7 mm (**a**), 5 mm (**b**) and 3 mm (**c**). The PTV D_98%_ decreased for each patient for all PTV margins evaluated

For the one fraction evaluated, the mean difference in CTV D_min,_ PTV D_95%_, PTV D_98%_, rectum D_15%_ and bladder D_mean_ between D1 and D2 was negative for the study population for all three PTV margins (Table 2). For the plans with 7 mm PTV margin, a decrease in D_min_ between D1 and D2 of the CTV was present in 15 patients, with one patient having a decrease larger than 2%. For the 5 mm PTV margin 18 patients had a decrease in D_min_, with four patients having a decrease of 2% or higher. When using a 3 mm PTV margin, there was a decrease in CTV D_min_ for 33 patients, with 11 patients having a decrease larger than 2% (Fig. 4). No statistically significant difference was found for CTV D_min_ between D1 and D2 for either the 7 mm PTV margin (*p* = 0.72) or the 5 mm PTV margin (*p* = 0.23). However, there was a significant difference in CTV D_min_ between D1 and D2 for 3 mm PTV margin (*p* < 0.01). The PTV D_98%_ decreased for every patient regardless of PTV margin and there was a statistically significant difference in PTV D_98%_ between D1 and D2 for all of the three PTV margins evaluated (Fig. 5). There was also statistically significant difference between D1 and D2 for the bladder. However, no statistically significant difference was found between D1 and D2 for the rectum for any of the PTV margins (Table 2).

**Table 2:** Dose difference between D1 and D2 for the evaluated PTV margins

	7 mm PTV margin [%]	5 mm PTV margin [%]	3 mm PTV margin [%]
CTV D_min_ *p*	−1.1 [−37.1 – 0.50] 0.72	−2.0 [−52.5 – 0.42] 0.23	−4.2 [−73.6 – 0.21] < 0.01
PTV D_95%_ *p*	−2.8 [−36.4 – 0.06] < 0.01	−2.9 [−39.2 – 0.09] < 0.01	−3.1 [−42.4 – 0.07] < 0.01
PTV D_98%_ *p*	−5.7 [−52.7 – −0.11] < 0.01	−5.2 [−51.5 – −0.26] < 0.01	−5.0 [−53.6 – −0.06] < 0.01
Rectum D_15%_ *p*	−3.6 [−52.3 – 28.7] 0.10	−3.6 [−56.1 – 41.7] 0.16	−2.6 [−56.0 – 43.0] 0.23
Bladder D_mean_ *p*	−12.6 [−32.2 – 13.5] < 0.01	−11.8 [−32.1 – 17.1] < 0.01	−10.2 [−32.7 – 26.7] < 0.01

The mean dose difference between D1 and D2 for CTV D_min_, PTV D_95%_, PTV D_98%_, rectum D_15%_ and bladder D_mean_. Values in square brackets represent the range of dose difference. Differences between D1 and D2 with PTV margin of 7 mm, 5 mm and 3 mm are shown. Negative numbers indicate a decrease in dose from D1 to D2. The *p*-value for each structure is displayed for all PTV margins

**Fig. 6:**
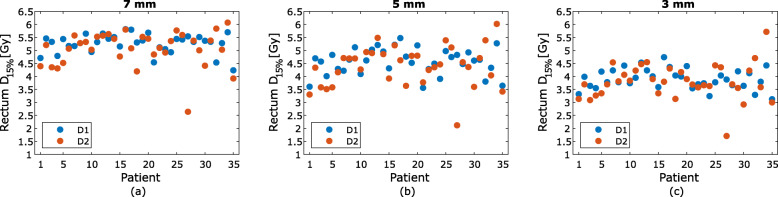
The rectum D_15%_ dose for the dose distributions D1 (blue) and D2 (orange) for PTV margins 7 mm (**a**), 5 mm (**b**) and 3 mm (**c**)

The dose to the rectum varied with the patients’ different motion patterns. Because of the increased distance between rectum and target for the smaller PTV margins of 5 mm and 3 mm, the corresponding treatment plans generally resulted in a lower rectum dose than for the 7 mm margin plans (Fig. 6). For 21 patients, there was a decrease in D_15%_, regardless of PTV margin. The dose to the bladder was below the constraint for all treatment plans.

## Discussion

This study investigated anatomical changes and prostate motion that occurred during a time frame corresponding to reported daily adaptive replanning time of 20 – 40 min in MR-linac systems [20–23], as well as the dosimetric consequences for one fraction, following these changes. This is to our knowledge the first study to relate the dosimetric effects of anatomical change and prostate displacements in localized prostate cancer patients, to the prolonged adaptive process time in an MR-linac workflow.

From our results it is evident that there is a variety in bladder volume increase, which indicate a different rate of bladder filling for different patients. The largest posterior prostate shifts were caused by large bladder volume increase, often in combination with muscle relaxation. Regarding the rectum, there was both an increase and decrease in volume. The largest increase in rectal volume was due to gas cavities, which either increased in size from MR1 to MR2 or appeared in MR2. These gas cavities caused the largest prostate shifts in the anterior direction. Our observations show that bladder and rectum volume changes are probable and individual and are therefore important processes to monitor during the adaptive replanning in the MR-linac. A possible approach for controlling the bladder volume could be to use a bladder filling protocol. The bladder volume would then initially be approximately the same at the start of every fraction. However, during the adaptive replanning process, there will still be a volume increase.

Prostate motion observed in this study, agrees well with results from previous studies [14, 15, 17]. The probability of prostate motion increases over time [13–18]. For example, Nederveen et al. [12] identified prostate displacements larger than 3 mm within only 3 min and Langen et al. [15] found that with time, the prostate tends to drift. The average prostate tracking time studied was 10 min and during this time prostate drifts larger than 10 mm occurred. Langen et al. further reported that with this motion pattern the prostate does not revert to its original position. Ballhausen et al. [17] also found a similar drifting motion of the prostate during 15 min and concluded that it is beneficial to reduce the treatment session duration. Cramer et al. [16] suggested a repositioning of the patient if the treatment session exceeded 4–6 min. These studies all observed prostate motion during a period frame substantially less than the time spent on adaptive replanning in an MR-linac.

Nejad-Davarani et al. [19] studied the dosimetric effect of anatomical changes in prostate cancer patients for an MRI-only workflow. MR images of volunteers with both full and empty bladder were acquired, with 45 min apart. A reference plan of the full bladder case was recalculated onto the empty bladder images. PTV D_95%_ was on average reduced by 11.5% when comparing the empty bladder treatment plans to the full bladder plans. The decrease in PTV D_95%_ was caused by prostate displacements in the anterior direction. Although Nejad-Davarani et al. used a bladder filling protocol with the full bladder condition as reference, the study indicates that differences in bladder filling can cause prostate shifts large enough to significantly decrease the dose coverage to the target, which is consistent with our findings. Nejad-Davarani et al. also found a larger reduction of dose to the target than we did in this study, which may be due to their longer separation between image acquisition.

Accordingly, with previous results and new data presented in this study, one can conclude that there is a considerable risk of prostate displacement during the adaptive process in the MR-linac. Before beam on, it will be crucial to verify the patient and prostate position thoroughly. This becomes especially important if imaging during treatment delivery is not available. According to our results, when using a PTV margin of 3 mm, there is a 20.0% risk of having to repeat adaptive replanning because of prostate motion larger than 3 mm. This indicates that a PTV margin of 3 mm might be too small to use while the adaptive replanning takes 20–40 min in the MR-linac workflow, as it currently does.

Furthermore, the treatment delivery time when using an MR-linac system for ultra-hypofractionation of the prostate can take up to 15 min [23]. This means that from that the irradiation begins the prostate has probably started to drift and this drifting motion will continue throughout the extended treatment delivery. Even if imaging throughout treatment delivery is used and translational couch shifts are applied based on prostate position, there could be negative dosimetric impacts on the OARs because of further deformation of these.

There are of course many possible benefits of using an MR-linac instead of a conventional linac for prostate cancer patients. The main advantages are the soft tissue contrast of the MR images, the ability of imaging during irradiation without additional dose and the possibility of adaptive treatment [28, 29]. However, the prolonged time for adaptive replanning and treatment delivery of the MR-linac is a drawback, which increases the risk of a target underdosage unless the anatomical changes are taken into account. On the MR-linac there is the possibility of acquiring images during the daily replanning and/or right before beam on. Adjusting the plan or patient position based on these images would likely reduce underdosage to the target. However, position verification imaging before treatment start must be thorough. As shown in this study, prostate motion can occur in all directions because of various anatomical changes and a two dimensional (2D) image in one or two planes might not be sufficient to detect motion causing target underdosage, especially when using a 3 mm PTV margin.

The dose to the rectum was in this study optimised according to the local dose protocol which is valid for a PTV margin of 7 mm. However, it is not entirely accurate for the treatment plans with 5 and 3 mm PTV margins. If the locally used PTV margin of 7 mm is changed to 5 or 3 mm, it is highly likely that the dose criterion for the rectum also would be changed. The comparison between D1 and D2 using the local DVH criteria was performed in order to clearly show that with a smaller PTV margin the rectum dose could be reduced. The dose to the rectum was nonetheless decreased for D2, regardless of PTV margin. Rectum is one of the most important OARs to consider when planning the treatment for prostate cancer patients. Therefore, a decrease in rectal dose can appear positive, since the side effects might be reduced. However, the observed results in this study demonstrate that this would be at the expense of an impaired dose coverage of the prostate.

All treatment plans in this study were planned using VMAT. VMAT is not yet available on the MR-linac unit, instead intensity modulated radiotherapy (IMRT) is the available delivery technique. Tetar et al. [23] reported a use of 15 field-IMRT for their prostate cancer patients, which generated a similar dose distribution as for a VMAT treatment plans, which justifies our method using VMAT plans in this evaluation. Since VMAT treatment plans provides a more conform dose distribution and a faster delivery time [30], VMAT will likely be clinically available for MR-linacs in the future.

A limitation of this study is that it is not performed on an MR-linac. However, the purpose of this study was to quantify anatomical changes that can occur during the duration of adaptive replanning. This was achieved by acquiring MR images on a conventional MR camera and thereafter calculate the dose on sCTs, without the need of an MR-linac system. Anatomical changes and dosimetric effects were evaluated for one fraction only in this study. This approach required the least amount of assumptions of the patient’s motion pattern, since only one pair of images was available for each patient. It is not likely that a patient moves in the exact same pattern with the same amount of deformation in rectum and bladder throughout all seven fractions. One option could have been to add the dosimetric impact from one fraction to the remaining six, with the assumption that during these six fractions, treatment is delivered exactly as planned. This scenario was also considered unlikely. A future extension to this study will be to acquire MR images of volunteers, during multiple days. More images with less time in between could be acquired during the same imaging session. Another possibility could be to use a 4D MR sequence. With more images acquired during multiple imaging sessions it could be possible to assess if a patient’s motion pattern is similar or differs from day to day. It would also be possible to evaluate whether the bladder fills at the same rate and how much the rectal activity can differ between fractions. This would enable investigation of how beneficial a bladder filling protocol or dietary protocol could be for individual patients. In addition, the motion of the prostate can be studied to examine if displacements mainly occur early, late or evenly throughout the treatment session. The distribution between sudden shifts and slow drifting motion could also be evaluated. Such information could be useful when choosing between conventional radiotherapy and treatment on an MR-linac.

The MR-linac systems are constantly evolving and the MR-linac workflow will most likely become faster as development continues. However, until a faster daily adaptive replanning process is possible, this study could help raise awareness about the possible limitations of treating prostate cancer patients with an MR-linac and underline that there are potential risks and disadvantages for this specific category of patients.

## Conclusion

Adaptive treatment including online non-ionising imaging and replanning is certainly the way forward for advanced personalized radiotherapy. However, there is a need for speed in the current MR-linac workflow to be able to shrink the conventional prostate cancer treatment margin. We have shown that there is a significantly increased risk of target underdosage in the adaptive replanning MR-linac workflow, due to anatomical changes in prostate cancer patients. Volume changes large enough to displace the prostate were seen for both the bladder and rectum. Even so, when faster workflows are available on the MR-linac, its full potential can be used and is likely to be the preferred treatment technique for hypofractionated prostate cancer patients.

## Data Availability

The datasets generated during/or analyzed during the current study are not publicly available due to patient privacy concerns and institutional regulations.

## Abbreviations

EBRT: External beam radiotherapy
PTV: Planning target volume
CT: Computed tomography
MR: Magnetic resonance
MRI: Magnetic resonance imaging
sCT: synthetic computed tomography
MR-linac: Magnetic resonance linear accelerator
Min: Minutes
FOV: Field of view
CTV: Clinical target volume
OARs: Organs at risk
FFF: Flattening filter free
VMAT: Volumetric modulated arc therapy
DVH: Dose volume histogram
TPS: Treatment planning system
MU: Monitor units
CoM: Center of mass
AP: Anterior-posterior
CC: Cranio-Caudal
LR: Left-right
IMRT: Intensity modulated radiotherapy
2D: Two dimensional
